# Injectable Click Polypeptide Hydrogels via Tetrazine-Norbornene Chemistry for Localized Cisplatin Release

**DOI:** 10.3390/polym12040884

**Published:** 2020-04-10

**Authors:** Zhen Zhang, Chaoliang He, Xuesi Chen

**Affiliations:** 1 CAS Key Laboratory of Polymer Ecomaterials, Changchun Institute of Applied Chemistry, Chinese Academy of Sciences, Changchun 130022, China; zzhang@ciac.ac.cn; 2 University of Science and Technology of China, Hefei 230026, China

**Keywords:** injectable hydrogel, polypeptide, click chemistry, local cisplatin delivery

## Abstract

Injectable, covalently cross-linked hydrogels have been widely investigated in drug delivery systems due to their superior mechanical properties and long-term stability. Conventional covalently cross-linked hydrogels are formed by chemical reactions that may interfere with natural biochemical processes. In this work, we developed an injectable polypeptide hydrogel via an inverse electron demand Diels-Alder reaction between norbornene modified poly(L-glutamic acid) (PLG-Norb) and tetrazine functionalized four-arm poly(ethylene glycol) (4aPEG-T) for localized release of cisplatin (CDDP). The rapid and bioorthogonal click reaction allowed for hydrogel formation within a few minutes after mixing the two polymer solutions in phosphate buffer saline (PBS). Dynamic mechanical analysis suggested that the storage modulus of the hydrogel could be readily tuned by changing the polymer concentration and the molar ratio of the two functional groups. The carboxyl groups of PLG-Norb were used to form polymer–metal complexation with CDDP, and the controlled release of the antitumor drug was achieved in PBS. The CDDP-loaded hydrogel displayed an antitumor effect against MCF-7 cells in vitro, through S phase cell cycle arrest. After subcutaneous injection in rats, the hydrogel was rapidly formed in situ and showed good stability in vivo. In an MCF-7-bearing nude mice model, the CDDP-loaded hydrogel exhibited an improved antitumor effect with reduced systemic toxicity. Overall, the injectable click polypeptide hydrogel shows considerable potential as a platform for localized and sustained delivery of antitumor drugs.

## 1. Introduction

Cisplatin (CDDP) is one of the first-line chemotherapeutic agents for the treatment of various types of epithelial malignancy, including head and neck, lung, ovarian, bladder and testicular cancer. The major mechanism of action of CDDP has been linked to its ability to covalently bind to the purine bases on the DNA, forming DNA–platinum adducts. This activates multiple signal transduction pathways involved in cell cycle arrest, DNA damage repair, and apoptotic cell death [1,2]. However, its clinical outcomes are compromised by severe side effects such as dose-limiting nephrotoxicity, gastrointestinal toxicity, peripheral neuropathy, and ototoxicity [3]. To address these issues, controlled drug delivery systems [4], including micelles [5], liposomes [6] and hydrogels [7], have been developed in recent decades to realize the higher drug concentration at tumor sites and the controlled release profile for a prolonged period of time.

Hydrogels, a class of cross-linked polymer networks retaining a large amount of water, possess interconnected porous structures and tunable mechanical properties. The high water content and viscoelastic nature of hydrogels provide compatibility with most living tissues, thus making them specifically intriguing for drug release, tissue engineering, and soft electronics [8,9,10]. In particular, injectable hydrogels have been developed that transform from liquid precursor solutions to solid-like hydrogels once injected into the body [11,12]. They can adapt to the shape of the injection site and form strong interfaces with the surrounding tissue. When acting as drug delivery systems, injectable hydrogels can be readily mixed with drugs outside the body and gel in situ, thus leading to the formation of drug depots for localized and sustained release.

Injectable hydrogels can be formed by physical interactions or covalent bonds [13,14,15]. For instance, in situ-forming hydrogels based on the block copolymers of poly(ethylene glycol) (PEG) and poly(*γ*-ethyl-L-glutamate) (PELG) were designed for the localized delivery of antitumor drugs. With the increase in temperature, the aqueous solution of the block copolymers could undergo sol-gel phase transition due to the formation of an aggregation network from amphiphilic PEG-PELG micelles [16,17]. Although the physical hydrogels can be formed under particularly mild conditions, they are usually mechanically weak and exhibit low long-term stability. Alternatively, covalent cross-linking strategies, such as radical polymerization, azide-alkyne cycloaddition, Diels-Alder reaction, and hydrazone and oxime ligation, have been exploited to overcome the challenges related to physical hydrogels [18,19]. Many of these covalent cross-linking reactions produce stable hydrogels with tunable mechanical properties, but they may not be optimal for biomedical applications due to the involvement of cytotoxic catalysts and the cross-reactivity with functional groups such as thiol and amine [20]. Recently, the inverse electron demand Diels-Alder (iEDDA) click reaction between tetrazine and olefin has emerged as a promising cross-linking strategy due to its exceptionally fast kinetics, excellent bioorthogonality and biocompatibility [21,22]. Upon mixing the clickable polymer solutions, hydrogels formed within minutes without any initiators, catalysts, or external energy input, allowing cell encapsulation and in vivo injection [23,24,25,26].

In the current study, an injectable polypeptide hydrogel based on iEDDA reaction was designed for localized CDDP delivery. The injectable and in situ-forming hydrogels were prepared via iEDDA reaction between norbornene modified poly(L-glutamic acid) (PLG-Norb) and tetrazine functionalized four-arm PEG (4aPEG-T). The gelation kinetics and mechanical properties of different hydrogel formulations were investigated by rheometer. CDDP was incorporated in the hydrogels by the complexation with carboxyl group of PLG-Norb. The in vitro cytotoxicity and cell cycle arrest were evaluated against MCF-7 cells. The in vivo antitumor efficacy was examined in BALB/c nude mice bearing MCF-7 xenografts by a single peritumoral injection of CDDP-loaded hydrogels.

## 2. Materials and Methods

### 2.1. Materials

5-Norbornen-2-methanol (mixture of isomers), *N*-hydroxysuccinimide (NHS), and isopentyl nitrite were purchased from TCI (Tokyo, Japan). 1-(3-Dimethylaminopropyl)-3-ethylcarbodiimide hydrochloride (EDC·HCl), 4-dimethylaminopyridine (DMAP), 4-cyanobenzoic acid, and pyrimidin-2-carbonitrile were purchased from J&K (Beijing, China). Four-arm PEG (number average molecular weight: 10,000 g/mol) was purchased from Pharmicell (Seoul, South Korea). *N*,*N*-Dimethyl formamide (DMF), dimethyl sulfoxide (DMSO), and pyridine were distilled under reduced pressure with calcium hydride. The deionized water was produced by a Milli-Q Water purification system (Millipore Co., Burlington, MA, USA).

### 2.2. Methods

^1^H NMR spectra were collected on an Avance 300 NMR spectrometer (Bruker, Billerica, MA, USA). The molecular weight and polydispersity index (PDI) were measured by gel permeation chromatography (GPC) (Waters, Milford, MA, USA). The eluent was 0.2 M phosphate buffer (PB) (pH 7.4) or DMF. Monodispersed PEG standards (Sigma) were used for the calibration curve. The circular dichroism (CD) spectra were obtained on a Chirascan spectrometer (Applied Photophysics, Leatherhead, Surrey, UK) at polymer concentration of 0.01 mg/mL in 5 mM PB (pH 7.4).

### 2.3. Synthesis of PLG-Norb

The poly(L-glutamic acid) (PLG) was prepared by ring-opening polymerization of *γ*-benzyl-L-glutamate *N*-carboxyanhydride, followed by deprotection. The PLG-Norb was prepared by coupling PLG with 5-norbornen-2-methanol by EDC·HCl and DMAP. Briefly, PLG (0.50 g) was dissolved in DMF (40 mL), and EDC·HCl (89 mg, 0.46 mmol), DMAP (5.7 mg, 0.047 mmol) and 5-norbornen-2-methanol (29 mg, 0.23 mmol) were added. The solution was stirred for 3 days at 23 °C. The product was dialyzed against deionized water for 3 days and lyophilized.

### 2.4. Synthesis of 4aPEG-T

4-Cyanobenzoic acid (7.4 g, 50 mmol) and pyrimidin-2-carbonitrile (6.3 g, 60 mmol) were suspended in ethanol (30 mL) and hydrazine monohydrate (12 mL, 250 mmol) was added dropwise. Afterwards, the mixture was heated to 90 °C and stirred for 10 h. After cooling down, the mixture was filtered. The solid was heated in acetone (50 mL) to reflux and filtered. The solid was then suspended in acetic acid (100 mL), followed by slow dropwise addition of isopentyl nitrite (12 mL). After stirring overnight, the purple product was precipitated in ethyl ether (200 mL). Subsequently, the mixture (3.3 g) was suspended in DMF (100 mL), and NHS (2.7 g, 23 mmol) and EDC·HCl (4.5 g, 23 mmol) were added. The mixture was stirred overnight at 40 °C. After that, DMF was removed under reduced pressure. The residue was dissolved in CH_2_Cl_2_, washed three times with water, and dried over Na_2_SO_4_. The 2,5-dioxopyrrolidin-1-yl 6-(6-pyrimidin-2-yl)-1,2,4,5-tetrazin-3-yl)benzoate (T-NHS) (2.2 g, 5.8 mmol) was purified by silica gel column chromatography (CH_2_Cl_2_/EtOAc 3:1) as a red crystalline solid.

The clickable 4aPEG-T macro cross-linker was synthesized by coupling T-NHS to four-arm PEG with amino end groups (4aPEG-NH_2_). To DMSO/pyridine (19:1, v/v, 20 mL), 4aPEG-NH_2_ (2.0 g, 0.20 mmol) and T-NHS (0.60 g, 1.6 mmol) were added. The solution was stirred for 48 h at 50 °C. DMSO was evaporated in vacuo and the mixture was redissolved in CH_2_Cl_2_ (100 mL), filtered, washed with brine (3×30 mL) and dried by Na_2_SO_4_. The 4aPEG-T was purified by silica gel column chromatography (CH_2_Cl_2_/methanol, 5:1) and precipitated in diethyl ether as a pink powder.

### 2.5. Preparation of Hydrogels

PLG-Norb and 4aPEG-T polymers were separately dissolved in PBS (10 mM, pH 7.4) to the desired concentration (3–5% (w/v)). Click hydrogels were prepared by simply mixing PLG-Norb and 4aPEG-T solutions at various norbornene/tetrazine molar ratios.

### 2.6. Dynamic Mechanical Analysis and Morphology of Hydrogels

For gelation kinetics measurement, PLG-Norb and 4aPEG-T solutions were mixed at a desired ratio and immediately pipetted onto the bottom plate of a US 302 Rheometer (Anton Paar, Graz, Austria), with a flat upper plate (25 mm in diameter). The samples were sealed by silicon oil to prevent water evaporation. The storage modulus (G′) and loss modulus (G″) were monitored as a function of time at a strain of 1% and a frequency of 1 Hz. To investigate the morphology, cylindrical hydrogel disks (3% (w/v)) were immersed in liquid nitrogen, lyophilized, and then coated with gold. The interior morphology of hydrogels was observed by scanning electron microscope (SEM, Micrion, FEI, Hillsboro, OR, USA).

### 2.7. In Vitro Hydrogel Degradation

In vitro degradation of hydrogels was measured by remaining mass in a degradation medium. PLG-Norb and 4aPEG-T solutions (3% (w/v)) were mixed in vials to form hydrogel (10 mm in diameter, 5 mm in height). Three milliliters of PBS containing 5 U/mL elastase was added on the top of formed hydrogels as a degradation medium. The remaining mass was measured at a predetermined time.

### 2.8. CDDP-Loaded Hydrogels and In Vitro Release

CDDP was loaded in hydrogels by the complexation of carboxyl groups in PLG-Norb with platinum (Pt). Typically, 3% (w/v) PLG-Norb in deionized water (1 mL) was mixed with CDDP (4 mg) and then shook at 37 °C for 72 h in the dark. During complexation, CDDP gradually dissolved and the mixture became a clear solution. For CDDP-loaded hydrogels, the complex solution was mixed with the same volume of 3% (w/v) 4aPEG-T in 20 mM PBS. The in vitro release was performed in vials as described above and 3 mL PBS was added as the release medium. At given time intervals, the release medium was collected, and fresh PBS was then added. The release behavior in PB without NaCl was tested for comparison. The concentration of released Pt was determined by ICP-MS (X series II, ThermoScientific, Waltham, MA, USA).

### 2.9. Cell Culture

MCF-7 (Human breast adenocarcinoma cell line) was cultured in Dulbecco’s modified Eagle’s medium (DMEM, Gibco, Waltham, MA, USA) with 10% fetal bovine serum, 50 U/mL streptomycin and 50 U/mL penicillin at 37 °C in a 5% CO_2_ atmosphere.

### 2.10. In Vitro Cytocompatibility

In vitro cytocompatibility of the PLG-Norb and 4aPEG-T were assessed by MTT assay. MCF-7 cells were seeded in a 96-well culture plate (5×10^3^ cells per well) and incubated for 24 h, after which a threefold dilution series of 20 μL polymer solution in DMEM was added. The culture plate was incubated for 48 h, followed by adding 20 μL MTT solution (5 mg/mL) to each well. The absorbance value at 490 nm was detected on a microplate reader (ELx 680, BioTek, Winooski, VT, USA). Cell viability (%) was determined by comparing the absorbance values of the experiment and control wells.

### 2.11. In Vitro Antitumor Effect and Cell Cycle Analysis

MCF-7 cells were seeded in 24-well plates (2×10^4^ cells, 1.5 mL DMEM per well) and incubated for 24 h. Sixty microliters of CDDP-loaded hydrogel was added into Transwell^®^ insert. MTT assay was carried out after 24, 48 and 72 h. For cell cycle analysis, the cells were treated for 24 h and digested by trypsin, washed with PBS and fixed with 70% ice-cold ethanol overnight at −20 °C. After that, the cells were washed with PBS and resuspended in 100 μL RNase A working reagent for 30 min at 37 °C. Then, 400 μL propidium iodide (PI) working reagent was added and the cells were incubated for 30 min at 4 °C away from light before being analyzed by a flow cytometer (FCM, Beckman Coulter, Brea, CA, USA).

### 2.12. In Vivo Biocompatibility

In vivo biocompatibility of the hydrogels was evaluated in female Sprague-Dawley rats. All experiments were approved by the Animal Care and Use Committee of Jilin University and all animals received care according to the Guide for the Care and Use of Laboratory Animals. In brief, 0.3 mL hydrogel (3% (w/v)) was subcutaneously injected into the back of rats by a 1.0 mL syringe. At given time points, the rats were sacrificed and the hydrogels were imaged. The skin tissues surrounding the hydrogels were collected and processed by hematoxylin and eosin (H&E) and Masson’s trichrome staining to examine the inflammatory response to the hydrogels.

### 2.13. In Vivo Antitumor Efficiency

Female BALB/c nude mice (5–6 weeks, 18g) were purchased from Beijing HFK Bioscience. A human breast adenocarcinoma xenograft tumor model was established by subcutaneous injection of MCF-7 cells (0.1 mL, 1×10^6^ cells) in the right flank. Treatments were carried out when the tumors reached around 60 mm^3^. The mice were randomly divided into 4 groups (*n* = 5), and 100 μL of PBS, free CDDP, and CDDP-loaded hydrogels were subcutaneously injected beside the tumor by a 1.0 mL syringe. The tumor size was measured with vernier calipers every 2 days and the tumor volume was calculated according to V = a × b^2^/2, in which a and b are the length and width of the tumor, respectively. The inhibition rate was calculated as (V_c_ − V_t_)/V_c_ × 100%, in which V_c_ and V_t_ were the tumor volumes of control and treatment groups, respectively. The body weights of the mice in each group were also recorded to indicate the systemic toxicity. At the end of experiments, the tumors and main organs were fixed in 4% (w/v) paraformaldehyde, embedded in paraffin, cut into 5 μm slices and then stained with H&E.

### 2.14. Statistical Analyses

The statistical analysis was carried out by one-way ANOVA, and *p* values < 0.05 were considered to be statistically significant.

## 3. Results and Discussion

### 3.1. Synthesis and Characterization of PLG-Norb and 4aPEG-T

**Scheme 1:**
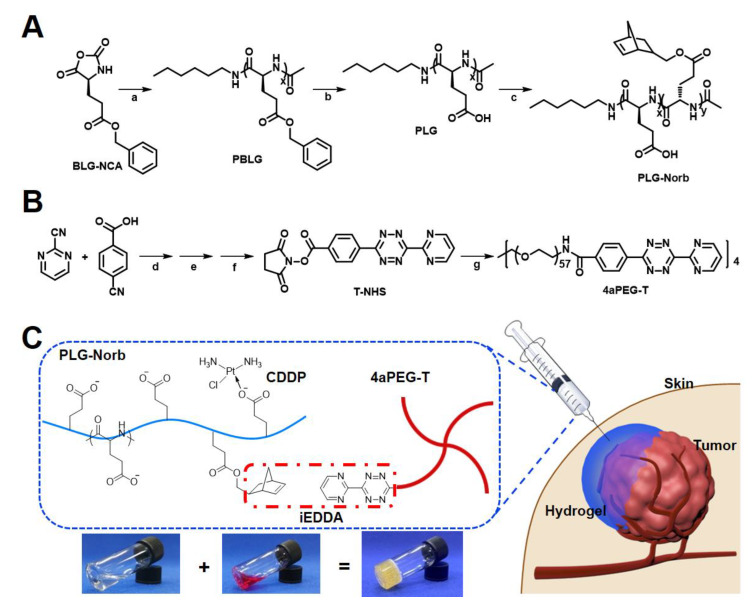
(**A**,**B**) Synthetic routes of norbornene modified poly(L-glutamic acid) (PLG-Norb) and four-arm poly(ethylene glycol) (4aPEG-T): a) hexylamine, *N*,*N*-Dimethyl formamide (DMF), r.t., 72 h; b) hydrobromic acid, dichloroacetic acid, r.t., 2 h; c) 1-(3-Dimethylaminopropyl)-3-ethylcarbodiimide hydrochloride (EDC·HCl), 4-dimethylaminopyridine (DMAP), DMF, r.t.,72 h; d) N_2_H_4_·H_2_O, ethanol, 90 °C, 10 h; e) isopentyl nitrite, acetic acid, r.t., overnight; f) EDC·HCl, *N*-hydroxysuccinimide (NHS), DMF, 40 °C, overnight; g) dimethyl sulfoxide (DMSO), 50 °C, 48 h. (**C**) Schematic illustration of injectable click polypeptide hydrogels for local treatment of tumor.

**Figure 1:**
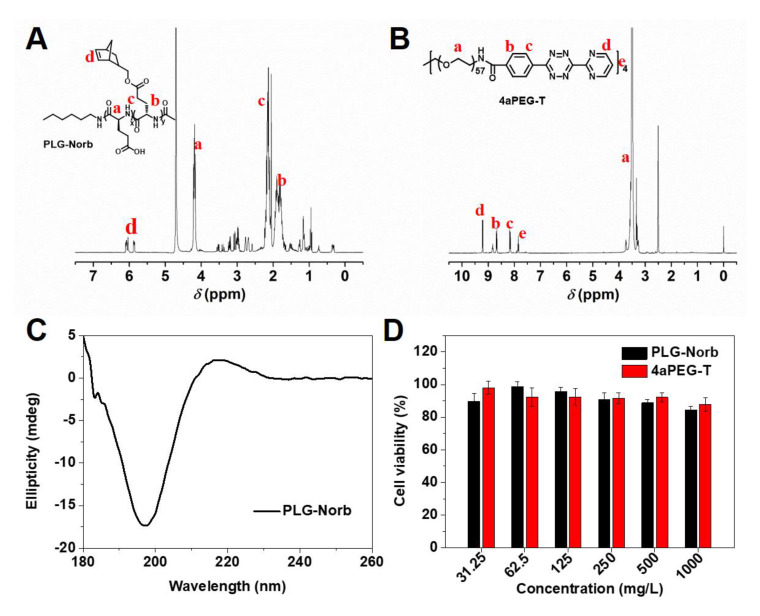
Representative ^1^H NMR spectra of (**A**) PLG-Norb and (**B**) 4aPEG-T using D_2_O/NaOD and DMSO-*d*_6_ as solvent, respectively. (**C**) Circular dichroism (CD) spectrum of PLG-Norb at 0.01 mg/mL in 5 mM PB (pH 7.4). (**D**) In vitro cytocompatibility of PLG-Norb and 4aPEG-T at different polymer concentrations against MCF-7 cells after incubation for 48 h (*n* = 6).

The synthetic strategy of PLG-Norb and 4aPEG-T is illustrated in Scheme 1. First, 5-norbornen-2-methanol was introduced to PLG through an esterification reaction. PLG is a well-accepted synthetic polymer for drug delivery owing to its good biocompatibility and degradability [27]. The *γ*-carboxyl groups of PLG are highly reactive and can be chemically modified to introduce various functional groups or to modulate the physicochemical properties of the polymer. A representative ^1^H NMR spectrum of PLG-Norb is presented in Figure 1A. The degree of substitution (DS) was calculated as 5.4% by the integration ratio of peaks for norbornene (*δ* 6.03, 5.99, and 5.83 ppm) to the peak for the PLG backbone (*δ* 4.14 ppm). The DS was close to the feed ratio of 6%, suggesting that the esterification reaction was very efficient in the presence of EDC·HCl and DMAP in DMF. The GPC curve of PLG-Norb exhibited unimodal distribution with PDI of 1.14. At pH 7.4, the PLG-Norb adopted a random coil conformation, as manifested by a negative minimum at 197 nm and a positive maximum at 217 nm in the CD spectrum (Figure 1C). This should be ascribed to nearly complete ionization of the carboxyl groups of PLG-Norb at pH 7.4. Additionally, a tetrazine group was introduced to the terminals of 4aPEG-NH_2_, yielding a clickable 4aPEG-T macro cross-linker with good water solubility. The average functionality of 4aPEG-T was determined to be 3.5 by the integration ratio of peaks for tetrazine (*δ* 9.22, 8.67, 8.15, and 7.85 ppm) to the peak for the PEG backbone (*δ* 3.51 ppm) (Figure 1B).

The in vitro cytocompatibility of PLG-Norb and 4aPEG-T polymers was evaluated by MTT assay. MCF-7 cells were incubated with PLG-Norb or 4aPEG-T at concentrations up to 1000 mg/L for 48 h. The relative cell viability is shown in Figure 1D. Over 90% of the MCF-7 cells exposed to all the polymer solutions remained viable, indicating no obvious cytotoxicity of the PLG-Norb and 4aPEG-T.

### 3.2. Preparation and Characterization of Copper-Free Click Polypeptide Hydrogels

To form click polypeptide hydrogels, PLG-Norb and 4aPEG-T polymer solutions in PBS were prepared separately. Upon mixing, spontaneous iEDDA reaction between tetrazine and norbornene groups occurred and free-standing hydrogels formed within a few minutes, a time-window that allowed injection through a needle. Meanwhile, the red color of the tetrazine group vanished and nitrogen gas generated from the cross-linking reaction led to the formation of micro-bubbles within the hydrogels.

**Figure 2:**
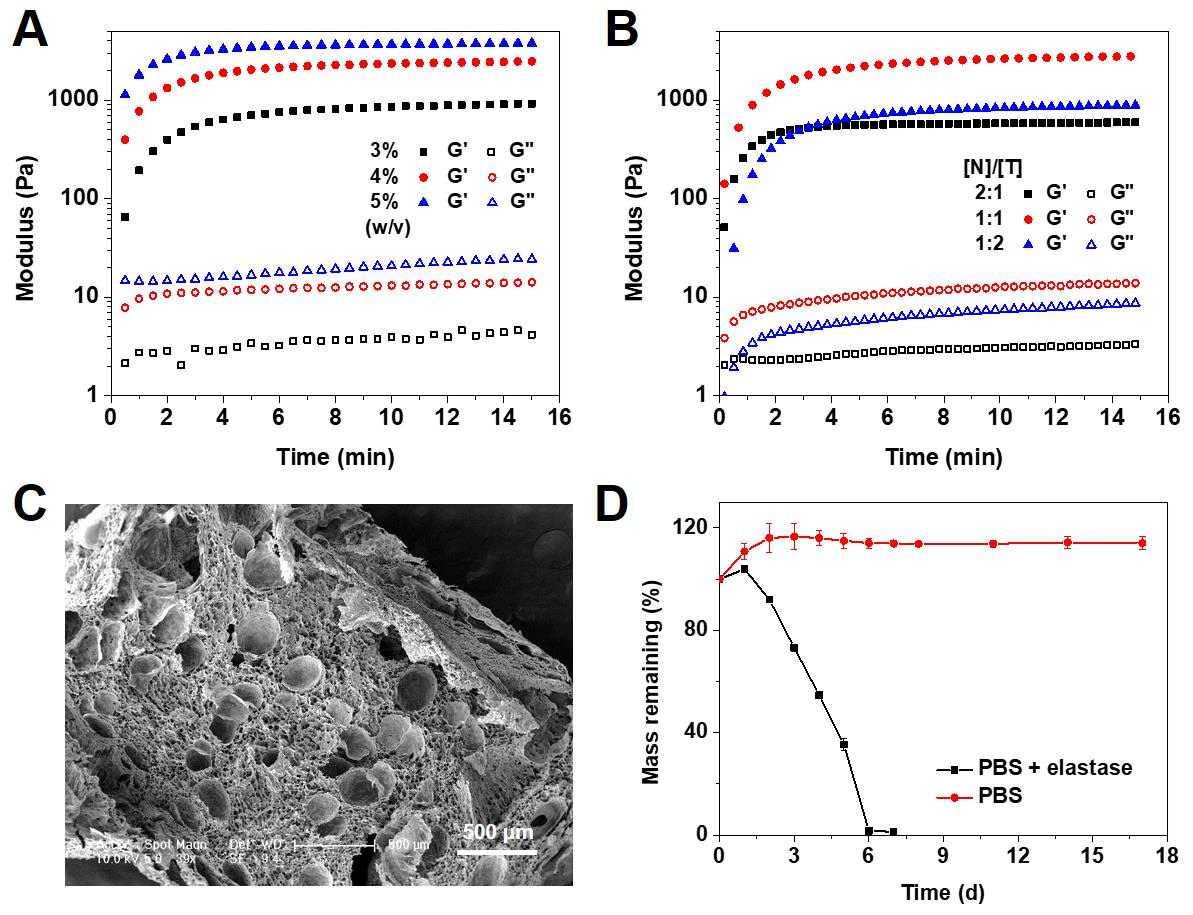
Storage modulus (G′) and loss modulus (G″) as a function of time for hydrogels (**A**) at polymer concentrations of 3%, 4%, and 5% (w/v) with [N]/[T] of 1:1 and (**B**) at a polymer concentration of 4% (w/v) with [N]/[T] of 2:1, 1:1, 1:2. (**C**) SEM images of the click polypeptide hydrogels. (**D**) In vitro degradation profiles of the hydrogels in PBS with or without 5 U/mL elastase.

To investigate the rheological behaviors in the process of cross-linking, the G′ and G″ of the hydrogels were monitored as a function of time. The G′ rapidly increased and then reached a plateau within 10 min, indicating approximate completion of the cross-linking reaction. The G′ of the hydrogels varied with the polymer concentration and the molar ratio of norbornene group to tetrazine group (denoted as [N]/[T]). In general, the G′ increased with the increase in polymer concentration. As shown in Figure 2A, the G′ changed from 0.70 to 3.8 kPa when the polymer concentration increased from 3% (w/v) to 5% (w/v). At a constant polymer concentration of 4% (w/v), the G′ could be altered from 0.6 to 2.8 kPa by adjusting the [N]/[T] (Figure 2B). Typically, the maximum mechanical strength was achieved at a [N]/[T] of 1:1, as an excess of norbornene or tetrazine group was unfavorable for the formation of intermolecular cross-linking [28]. SEM was employed to examine the inner morphology of the freeze-dried hydrogels. As shown in Figure 2C, the hydrogels possessed a highly porous network structure, which may be favorable for drug transport.

The degradation of polymer materials is a major concern for drug delivery systems. It is desirable that the hydrogels should degrade following drug depletion to avoid additional surgical removal of the carriers. For covalently cross-linked hydrogels, the degradation occurs through bond cleavage at the cross-links or in the polymer backbone, which is generally controlled by hydrolysis or enzyme catalysis [29]. The peptide bond in the PLG backbone is susceptive to the proteases widely distributed in the body. Hence, the in vitro degradation behavior of the hydrogels in PBS containing elastase was investigated by monitoring the remaining mass. As shown in Figure 2D, the mass of the hydrogels continuously decreased within 6 days, which should be attributed to the gradual breakdown of the PLG backbone and the consequent degradation of the polymer network. Without elastase, the hydrogels were quite stable in PBS, and the mass increased slightly due to the modest swelling of the hydrogels.

### 3.3. In Vitro Release from CDDP-Loaded Hydrogels

**Figure 3:**
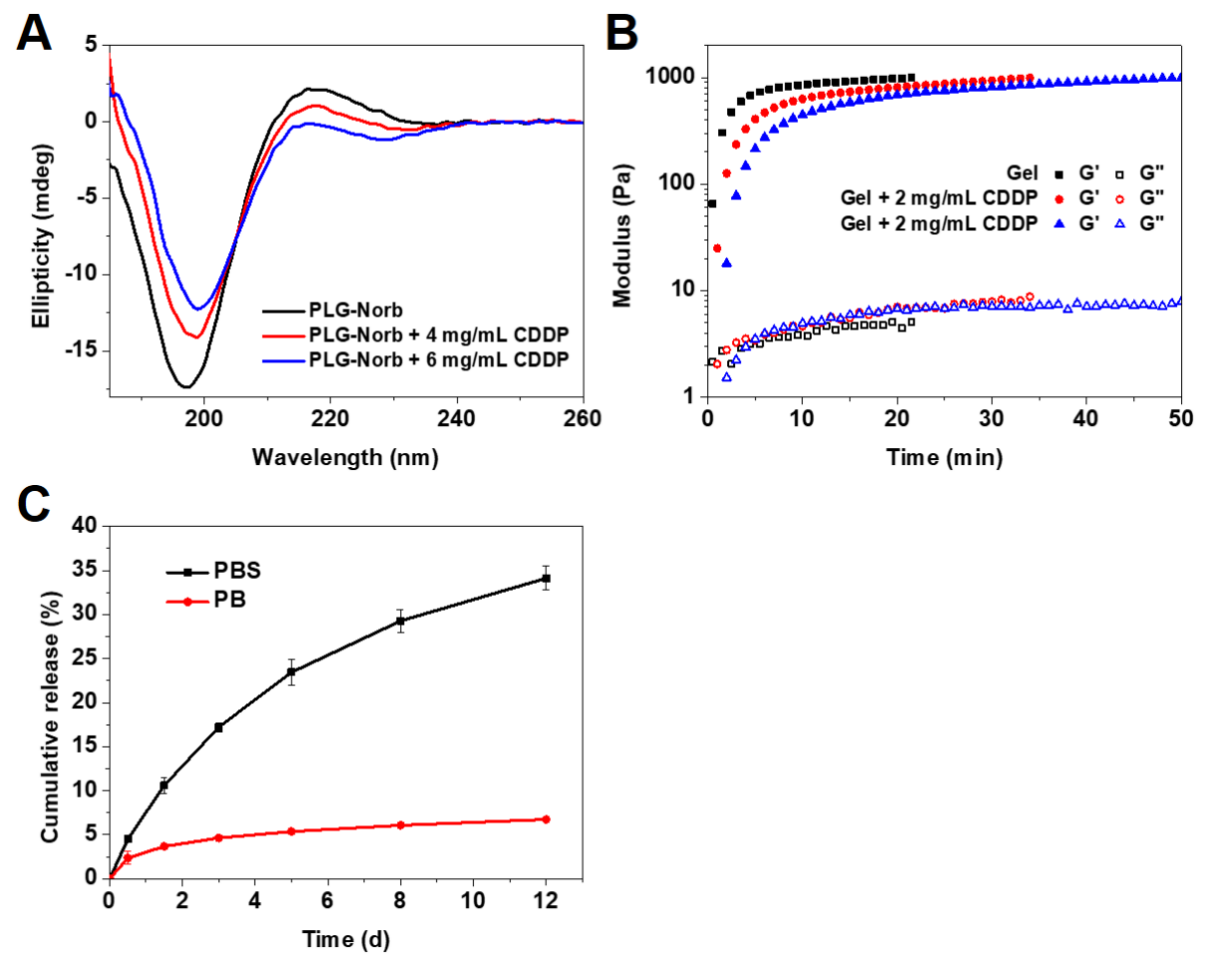
(**A**) CD spectra at the polymer concentration of 0.01 mg/mL in 5 mM PB (pH 7.4). (**B**) Gelation kinetics of cisplatin (CDDP)-loaded hydrogels with CDDP concentrations of 2 and 3 mg/mL. (**C**) In vitro release profiles of CDDP from the hydrogels in PBS or PB (without NaCl).

As the chloride leaving groups of CDDP can be substituted by carboxyl groups under low concentration of chloride ion, CDDP was loaded in the hydrogels by incubation with PLG-Norb in deionized water, followed by mixing with 4aPEG-T [5]. CD spectra revealed that binding of CDDP to carboxyl moieties of PLG-Norb induced a slight transition of secondary structure at pH 7.4 (Figure 3A). It was reported that PLG adopts a random coil conformation under neutral conditions, while it may form an *α*-helix structure with substitution in side chains because of the decreased intramolecular electrostatic repulsion [30,31]. Next, the complex solution was mixed with 4aPEG-T in 20 mM PBS to form hydrogels with a final concentration of 10 mM PBS. The iEDDA reaction between tetrazine and suitable dienophiles (e.g., norbornene, trans-cyclooctene) is reported to be fast and bioorthogonal, which makes the cross-linking process highly compatible with the encapsulated drugs, proteins and living cells [22]. The rheological tests for CDDP-loaded hydrogels demonstrated a decrease in gelation kinetics. As shown in Figure 3B, it would take a longer time for G′ to reach the same value when CDDP was bound to the PLG-Norb backbone as a result of the increased steric hindrance.

The in vitro release profile of CDDP from the hydrogels was evaluated in PBS at 37 °C. During the test period of 12 days, 34% of CDDP was released from the hydrogels in a controlled and sustained manner, due to the ligand exchange from carboxyl groups of PLG-Norb to chlorides (Figure 3C). In contrast, only 6.7% of CDDP was released from the hydrogels in PB without NaCl in 12 days.

### 3.4. In Vitro Tumor Cell Inhibition by CDDP-Loaded Hydrogels

**Figure 4:**
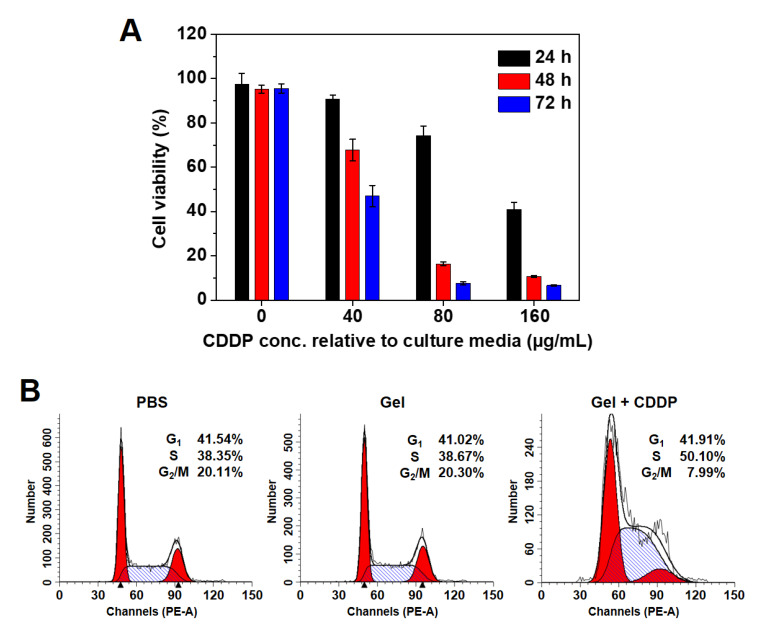
(**A**) In vitro cytotoxicity of CDDP-loaded hydrogels against MCF-7 cells. (**B**) Flow cytometric analysis of cell cycle distribution of MCF-7 cells induced by CDDP-loaded hydrogels.

The in vitro tumor cell inhibition efficiency of CDDP-loaded hydrogels was evaluated against MCF-7 cells. As shown in Figure 4A, the hydrogel itself exhibited no detectable cytotoxicity, with the cell viability remaining over 95%. In contrast, the CDDP-loaded hydrogels inhibited the proliferation of MCF-7 cells in a dose-dependent manner. After 24 hours of incubation, the cell viabilities reduced from 90.7% to 41.0%, as the final CDDP concentration relative to culture media augmented from 40 to 160 μg/mL. Increasing the incubation time further enhanced the antitumor effect of CDDP-loaded hydrogels, due to the sustained release of Pt complex. For example, at the CDDP concentration of 80 μg/mL, the cell viabilities were decreased to 74.2%, 16.4%, and 7.5% for 24, 48, and 72 h, respectively.

In addition, the effect of CDDP-loaded hydrogels on the cell cycle of MCF-7 cells was investigated by detecting DNA content after staining with PI. As illustrated in Figure 4B, CDDP-loaded hydrogels induced predominant S phase arrest of MCF-7 cells (50.10%) as compared with the PBS group (38.35%) [32,33,34], while the percentages of MCF-7 cells incubated with blank hydrogels in various phases were comparable to those of the PBS group. This further manifested the cytocompatibility of the iEDDA-based polypeptide hydrogels.

### 3.5. In Vivo Biocompatibility of Click Polypeptide Hydrogels

**Figure 5:**
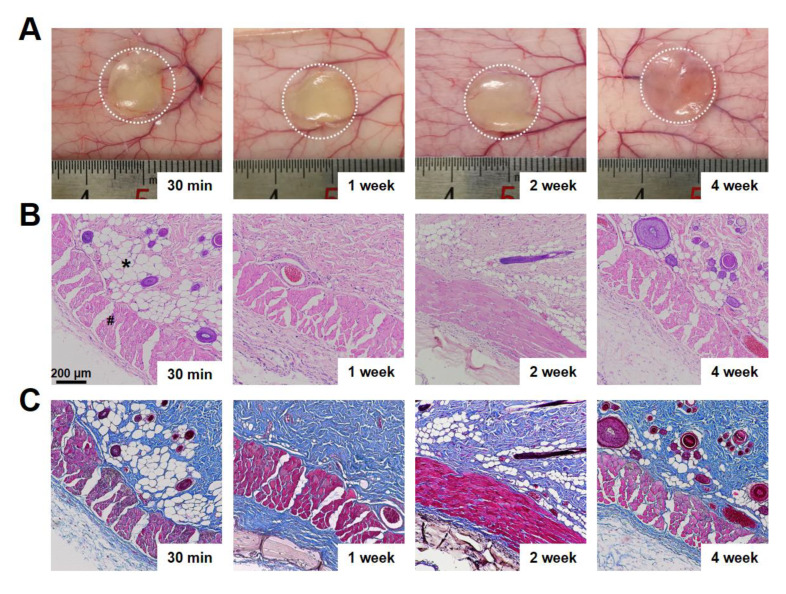
In vivo biocompatibility of click polypeptide hydrogels. (**A**) Representative images of hydrogels following subcutaneous injection into rats at different time intervals. (**B**) H&E staining, and (**C**) Masson’s trichrome staining of the hydrogels and the surrounding tissues at corresponding time intervals. The asterisks indicate the location of the hydrogels.

To investigate the in vivo biocompatibility of copper-free click polypeptide hydrogels, PLG-Norb and 4aPEG-T solutions in PBS were injected subcutaneously into the back of rats once mixed. At various time points, the rats were sacrificed and the injection sites were surgically dissected to expose the hydrogels with adjacent skin. It was observed that the hydrogels could form at the injection site without the extravasation into the surrounding tissues. Due to the stable linkages formed from the reaction between norbornene and tetrazine groups, the hydrogels maintained their structural integrity for 4 weeks and thin fibrous capsules were found to form around the hydrogels (Figure 5).

Furthermore, the host tissues surrounding the hydrogels were processed by H&E and Masson’s trichrome staining to evaluate the inflammatory response. One week after injection, increased inflammatory cells and collagen deposition were observed, suggesting an acute inflammatory response in the initial stage. After that, the inflammatory cells diminished gradually. Neither macroscopic edema, hyperemia, nor tissue necrosis were observed during the experiment. Thus, the results suggest that the hydrogels were well tolerated by the tissue and exhibited good histocompatibility.

### 3.6. In Vivo Antitumor Efficacy of CDDP-Loaded Hydrogels

**Figure 6:**
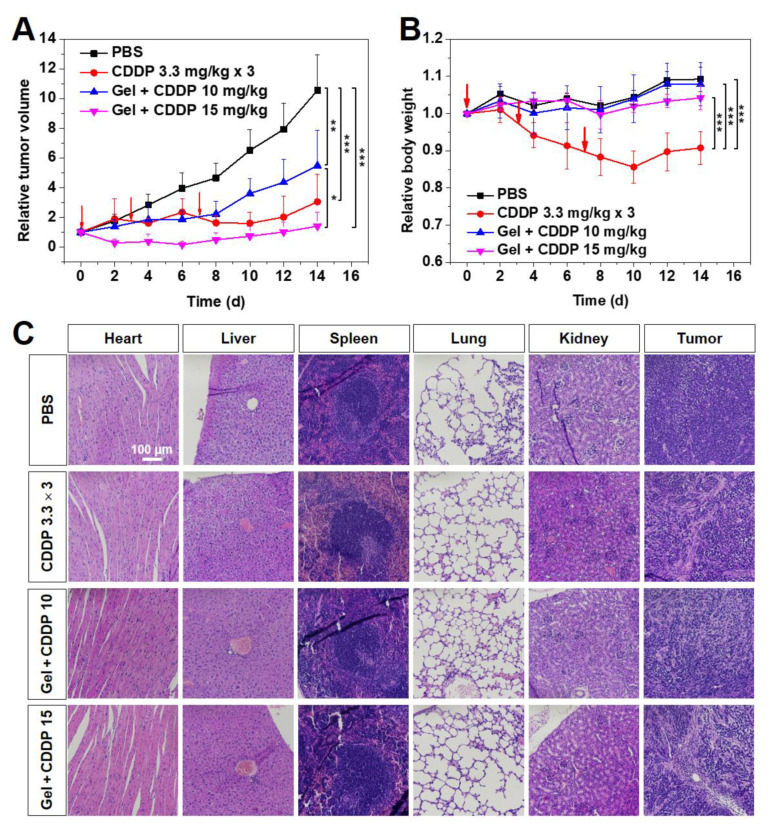
In vivo antitumor efficacy of CDDP-loaded hydrogels for treatment of BALB/c nude mice bearing MCF-7 xenografts. (**A**) Relative tumor volume. (**B**) Relative body weight. The arrows indicate 3 injections of free CDDP at each dose of 3.3 mg/kg on days 0, 3, 7. Data depict mean ± SD; values were analyzed by one-way ANOVA, * *p* < 0.05, ** *p* < 0.01, *** *p* < 0.001. (**C**) H&E staining of the tumor tissues and main organs of mice at day 14.

To evaluate the in vivo antitumor efficacy, BALB/c nude mice bearing MCF-7 tumors were treated by a single peritumoral injection of CDDP-loaded hydrogels at doses of 10 and 15 mg/kg, respectively. By comparison, mice received three peritumoral injections of free CDDP at each dose of 3.3 mg/kg on days 0, 3, 7, as mice were unable to tolerate a single injection of free CDDP at a dose of 10 mg/kg. As shown in Figure 6A, in the PBS group, the tumor size increased rapidly during the experiment. By comparison, the tumor growth was effectively inhibited in free CDDP and CDDP-loaded hydrogel groups. At the end of the treatment, the tumor inhibition rate (TIR) of CDDP-loaded hydrogels at a dose of 10 mg/kg was 48.1%, which was inferior to free CDDP (71.1%), likely as a result of incomplete release of CDDP from the hydrogels. It is noteworthy that the inhibitory effect of CDDP-loaded hydrogels could be further improved by increasing the dosage to 15 mg/kg, which showed a markedly enhanced TIR of 86.6%.

Moreover, the systemic toxicities of the treatments were assessed by monitoring the mouse body weights. It was shown that the mice treated with CDDP-loaded hydrogels retained stable body weights even if the CDDP dosage reached 15 mg/kg, indicating the low systemic toxicity of the hydrogel-based treatment (Figure 6B). However, treatment with free CDDP caused obvious (~10%) weight loss after the third injection. The reduced systemic toxicity of CDDP-loaded hydrogels compared with free CDDP should be mainly due to sustained release of CDDP at the tumor site.

Next, H&E staining was performed to evaluate the histological characteristics of the tumors and major organs in each group. As shown in Figure 6C, tightly packed tumor cells were observed in the tumor tissue of PBS group, suggesting a rapid tumor growth. After the treatment with chemotherapeutic agents, the cell density decreased and various degrees of necrotic areas were observed. On the other hand, no histopathological change was observed in the major organs from the animals of CDDP-loaded hydrogels group. This confirmed low toxic side effects of the treatment by localized and sustained release of CDDP from the hydrogels at the tumor site. In contrast, free CDDP induced obvious nephrotoxicity, as indicated by the shrinkage of renal capsule cavity [17].

## 4. Conclusions

In the present study, we developed an injectable polypeptide hydrogel via iEDDA reaction between PLG-Norb and 4aPEG-T for localized delivery of CDDP. The rapid and bioorthogonal click reaction allowed us to create hydrogels with a wide range of mechanical properties. The carboxyl groups of PLG were used to form a polymer–metal complexation with CDDP, leading to a sustained and prolonged release profile. For the in vivo treatment of mice bearing MCF-7 xenografts, a single peritumoral injection of the CDDP-loaded hydrogels exhibited significant antitumor efficacy and reduced systemic toxicity due to the localized, sustained CDDP release at the tumor site. Additionally, the click hydrogels have a minimal inflammatory response and good stability in vivo, making them attractive materials for long-term applications. Overall, the injectable click polypeptide hydrogel shows great potential as a platform for localized and sustained delivery of antitumor drugs.
